# A continuous-discrete model of cell contraction incorporating actin and intermediate filaments

**DOI:** 10.1016/j.isci.2026.116279

**Published:** 2026-06-05

**Authors:** Soheil Sarbishei, Yousef Javanmardi, Reza Azarbad, Morteza Naeij, Pradeep Keshavanarayana, Emad Moeendarbary, Fabian Spill

**Affiliations:** 1 School of Civil Engineering, College of Engineering, University of Tehran, Tehran 1439957131, Iran; 2 Department of Mechanical Engineering, University College London, Torrington Place, London WC1E 7JE, UK; 3 Cellular and Molecular Biology Research Center, Health Research Institute, Babol University of Medical Sciences, Babol 4717641367, Iran; 4 Department of Civil and Environmental Engineering, Amirkabir University of Technology, Tehran 158754413, Iran; 5 School of Mathematics, University of Birmingham, Birmingham B15 2TT, UK; 6 Centre for Computational Medicine, University College London, London WC1E 6JF, UK; 7 Department of Biological Engineering, Massachusetts Institute of Technology, Cambridge, MA 02139, USA

**Keywords:** Biological sciences, Biochemistry, Cell biology, Integrative aspects of cell biology, Functional aspects of cell biology

## Abstract

Cell contractility is driven largely by actin filaments (AFs), while intermediate filaments (IFs) contribute to mechanical stability and help maintain cellular architecture. Here, we present a continuous-discrete finite element model that represents AFs and IFs as one-dimensional elements embedded within a two-dimensional cell domain. AFs generate contractile forces, move toward regions of maximum mean strain, and align with the major principal strain, whereas IFs move toward regions of minimum mean strain and align with the minor principal strain. Model predictions agree with experimental measurements of membrane curvature and the spatial distribution of F-actin and IFs across cells cultured on different adhesion patterns and substrates. Simulations reveal that AFs accumulate near focal adhesions (FAs) and the plasma membrane and also assemble into cable-like bundles that cross the cytoplasm, while IFs resist excessive deformation and reduce membrane curvature. This framework provides a mechanistic approach for studying how cytoskeletal organization regulates cell contraction.

## Highlights

• A continuous-discrete model captures actin and intermediate filament dynamics
• Actin filaments accumulate near focal adhesions and the plasma membrane
• Intermediate filaments resist excessive deformation during cell contraction
• Model predictions agree with experimental membrane curvature measurements

## Graphical abstract

**Figure:**
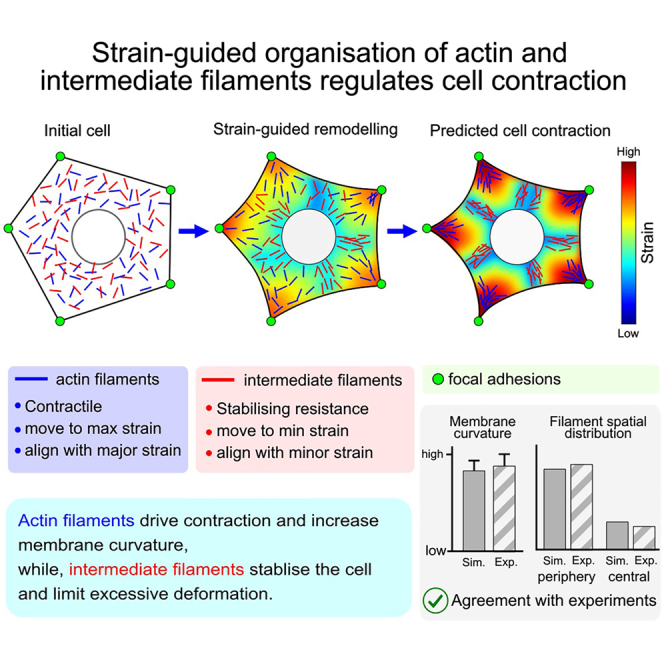

## Introduction

Cell contraction is essential for many biological processes, including adhesion,^1^^,^^2^ proliferation,^3^^,^^4^^,^^5^ locomotion,^6^^,^^7^^,^^8^ and T cell activation.^9^^,^^10^ The regulation of cell contraction is orchestrated by the cytoskeleton, a dynamic network of protein filaments that not only provides structural support to cells but also contributes to various cellular processes.^11^ Among the cytoskeletal proteins directly involved in cell contraction, actin filaments (AFs) play a crucial role^12^^,^^13^ through the formation of stress fibers (SFs).^14^^,^^15^ SFs particularly hold significance in generating traction forces.^16^^,^^17^^,^^18^ Additionally, intermediate filaments (IFs), including vimentin, desmin, and keratin, constitute another important group of cytoskeletal proteins that contribute to the mechanical strength and overall stability of the cell.^19^ While IFs are not as dynamic as AFs, they play a significant role in resisting against excessive cellular deformation.^20^

A range of mechanical frameworks has been devised to simulate cell contraction. These models can be categorized into four major classes: (*1*) Simple mechanical models grounded in concepts of surface tension and membrane stiffness, which offer intuitive insights and establish quantifiable parameters such as the radius of curvature.^21^^,^^22^ (*2*) Continuous models, varying in complexity from purely mechanical constructs that factor in active forces stemming from thermal strain, to bio-chemo-mechanical models that incorporate the biochemical intricacies of AFs to generate contractile forces within cells.^23^^,^^24^ (*3*) Discrete models employing one-dimensional spring elements to represent different cellular components, such as SFs and membrane, in which the springs’ constant controls cellular stiffness.^25^^,^^26^ (*4*) Continuous-discrete models, ranging from models based on regular elastic discrete elements, to complex configurations of discrete unidimensional AFs within a continuous cell domain.^14^^,^^27^^,^^28^^,^^29^

While simple mechanical models can offer insight into cell contraction and align with experimental observations, they do not account for AFs as distinct entities. On the other hand, the continuous modeling approach, incorporates the effects of AFs forces using equivalent active stress tensor in a continuum manner, neglecting the nature of AFs forces as concentrated dipole forces within the cell.^26^ Furthermore, numerous experimental studies have shown that AFs accumulate in specific locations near focal adhesions (FAs) during cell deformation,^30^ an observation typically not incorporated in continuum models.^23^^,^^24^ On the other hand, in a vast range of discrete and continuous-discrete models, AFs are treated as unidimensional entities exhibiting concentrated forces. In most of these studies, AFs were arranged in specific patterns within a cellular domain and cannot dynamically remodel and aggregate in specific locations.

Here, we present a finite element-based model employing the continuous-discrete approach, where AFs and IFs are stochastically distributed as one-dimensional elements within the cell. AFs, responsible for generating concentrated active forces, and IFs, contributing to the mechanical strength and maintaining cellular architecture, collectively influence the cellular deformation. In our model, AFs and IFs can actively reorient and reposition, enhancing the model’s ability to predict cell contractility.

## Results

The developed 2D finite element model incorporates several key features, outlined as follows.

1 AFs and IFs are regarded as one-dimensional elements.
2 AFs generate concentrated active dipole forces.
3 AFs and IFs undergo movement and reorientation in response to cellular deformation following a strain-based rule. In our model, this behavior is implemented through an imposed rule: AFs move toward adjacent points with the highest mean principal strain, while IFs move toward points with the lowest. Although this relocation rule is not derived from first principles, it is grounded in biological reasoning and enables the model to match experimental observations.

**Figure 1:**
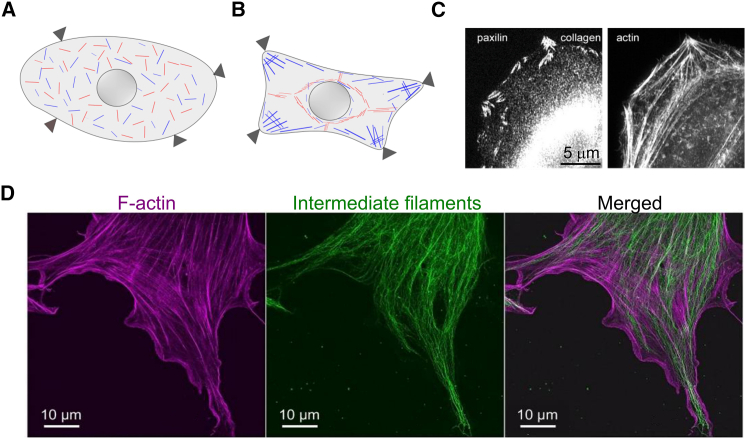
Model specifications (A) Schematic illustration of the model before deformation. Cytoplasm containing major (blue) and minor (red) fibers, representing actin filaments (AFs) and intermediate filaments (IFs), respectively. (B) Schematic representation of the deformed cell resulting from the forces exerted by AFs. (C) Arrangement of actin filaments near focal adhesions, cell cortex, and within cytoplasm (adapted from de Rooij et al.^31^). (D) Relative position of fibrous actin and vimentin intermediate filaments (adapted from Wu et al.^32^).

These features collectively contribute to capturing the behavior of AFs and IFs within the cell and their influence on cell deformation, providing insights into the dynamic nature of the cytoskeleton during cell adhesion processes. Figure 1A illustrates a schematic view of the model specifications.

### AFs/IFs as one-dimensional elements

Integrating one-dimensional AFs and IFs into a two-dimensional domain presents computational challenges, especially when using commercial finite element software, due to increased computational costs and the need for additional degrees of freedom. To address this issue, we borrowed a concept from the field of composite materials,^33^ which allows us to incorporate the effects of the one-dimensional filaments without adding extra degrees of freedom to the existing finite element mesh.

In our model, we explicitly assign the active concentrated dipole forces to individual AFs based on the formulation suggested by Deshpande et al.^23^ The cell is discretized using a planar 3-node triangular finite element mesh. The concentrated forces generated by the AFs are applied directly to the nodal points of the mesh through interpolation using shape functions (Methods S1). This approach enables us to realistically represent the active forces as concentrated point loads acting on the respective two-dimensional elements, eliminating the need to include any extra degree of freedom.

### Stochastic distribution of AFs/IFs within the domain

Experimental data have shown alterations in the assembly and orientation of the cytoskeletal components as the cell undergoes deformation.^34^^,^^35^ While the initial arrangement of the filaments may appear random, a discernible pattern emerges in their configuration during the cell adhesion process.^30^ Hence, we initially generate a set of AFs/IFs with random variation in both length and placement (Figure 1A). Subsequently, during the cell deformation process, these elements undergo elongation, shortening, rotation, or relocation within the domain based on specific rules (Figure 1B). These rules (Methods S1) aim to closely replicate the steady state filaments’ locations, orientation, and overall distribution observed in experiments.

### Movement of AFs/IFs in response to cell deformation

AFs can be found in various locations within cells. In particular, they are enriched near FAs^36^ and cell cortex,^37^ and are also present throughout the cytoplasm^38^ (Figure 1C). On the other hand, IFs reinforce the structural framework of cells, particularly in tissues subjected to mechanical stress or tension. They provide resistance against mechanical forces and help distribute stress throughout the cell, aiding in its mechanical stability.^39^ They can be observed extending throughout the cytoplasm, connecting to various cellular structures such as cell-cell junctions, cell-matrix adhesions, or the nucleus^19^ (Figure 1D).

Given that the AFs generate contractile force and therefore are stretched in the direction of the force, it is reasonable to anticipate having a local maximum in mean principal strains at both ends. Furthermore, we expect major principal strains to occur along the axial direction of these bundles. In contrast, IFs lie roughly perpendicular to the SFs^32^ (Figure 1D) and hence, it is reasonable to assume that they reorient in the direction of minor principal strains.

In our study, to simulate the impact of AFs and IFs on cellular contraction, we incorporate two types of 1-D filaments in our model, referred to as major and minor fibers, color-coded in blue and red, respectively. Major fibers are responsible for generating active compressive forces. They move toward adjacent points with the highest mean principal strain and rotate to align with the direction of the major principal strain. Minor fibers, on the other hand, contribute to structural stability by stiffening the cell. They move toward neighboring points with the lowest mean principal strain and reorient to align with the direction of minor principal strain (Methods S1).

To numerically implement these rules, the model evaluates the strain field in the neighborhood of each filament. Around both endpoints of a filament (denoted *A* and *B*), the surrounding finite elements are searched to identify the point with the highest (for AFs) or lowest (for IFs) mean principal strain. The relative displacement of each endpoint is then computed based on the strain gradient and spatial separation from this extremum. Specifically, the dislocation vectors at *A* and *B* are calculated as:

(Equation 1) $$\begin{array}{c} \Delta u_{A}=DF.d.\frac{{\bar{\varepsilon }}_{\max}-{\bar{\varepsilon }}_{A}}{RangeE}\\ \Delta u_{B}=DF.d.\frac{{\bar{\varepsilon }}_{\max}-{\bar{\varepsilon }}_{B}}{RangeE} \end{array}$$

where *DF* is a user-defined displacement factor controlling relocation speed, *d* is the distance between the endpoint and the strain extremum ${\bar{\varepsilon }}_{A}$ and ${\bar{\varepsilon }}_{B}$ are the local mean strains at the filament ends, and *RangeE* is the total range of mean strain values across the cytoplasmic domain. The overall displacement vector for the filament is then computed as the average of the two endpoint vectors and applied to shift the filament position accordingly.

Rotation is handled separately; the direction of major principal strain is evaluated at the midpoint of the filament and used to guide reorientation. Letting *θ* represent the current angle of the filament relative to the vertical axis, and *φ* the angle of the principal strain direction, the rotation angle is computed as:

(Equation 2) $$\Delta \theta =RF.\frac{\varphi -\theta }{Range\theta }$$

where *RF* is a rotation factor controlling angular responsiveness, and *Rangeθ* denotes the range of strain orientation angles throughout the domain. This step ensures that filaments gradually align with the principal strain directions over successive iterations. Full derivations and illustrations of these procedures are available in Methods S1 and Figures S4 and S5.

The relocation and orientation rules applied to actin and IFs in the model are biologically grounded and supported by both experimental data and theoretical predictions. AFs are known to dynamically reorganize in response to local mechanical strain, particularly near FAs and along the cell periphery. Several studies have shown that actomyosin SFs reorient along directions of the main principal strain under external mechanical stimuli such as stretch or variations in matrix stiffness.^32^^,^^40^^,^^41^

Mechanical simulations have shown that AFs under persistent contractive load aggregate and align in the direction of force, forming bundled SFs through force-induced reorganisation.^41^ These models demonstrate that external strain gradients can drive filament bundling and alignment, consistent with our imposed rule that AFs move toward high strain and align with the principal strain direction.

In contrast, IFs such as vimentin play a more passive, load-bearing role, contributing to mechanical resilience rather than active force generation. High-resolution microscopy and cryo-electron tomography studies have shown that vimentin forms interpenetrating networks with actin SFs, often wrapping around or running orthogonal to actin bundles.^32^ Additional work demonstrated that vimentin buffers mechanical loads across the cytoplasm, especially in regions of low actin density.^42^ These findings support the assumption that IFs accumulate in low-strain regions and align with the minor principal strain direction.

These experimental observations align with theoretical models that incorporate strain-dependent cytoskeletal dynamics. A bio-chemo-mechanical model demonstrated that local tension governs both the assembly and dissociation of SFs, which preferentially form in regions experiencing high strain.^23^ Further modeling studies established that strain-dependent reorientation of actin structures can occur to minimize mechanical perturbations.^43^ A recent finite element optimization study demonstrated that SFs self-organize to minimize intracellular stress, producing spatial configurations that closely resemble those observed experimentally in strained cells.^44^

While these rules are explicitly imposed rather than emergent in our implementation, they reproduce a well-established biological dichotomy: AFs behave as contractile, strain-aligned structures that promote force generation, while IFs act as deformable scaffolds that buffer and distribute mechanical loads. This modeling approach allows us to recapitulate the distinct mechanical behaviors and spatial organization of AFs and IFs observed across a wide range of experimental systems.

### Description of the model

**Figure 2:**
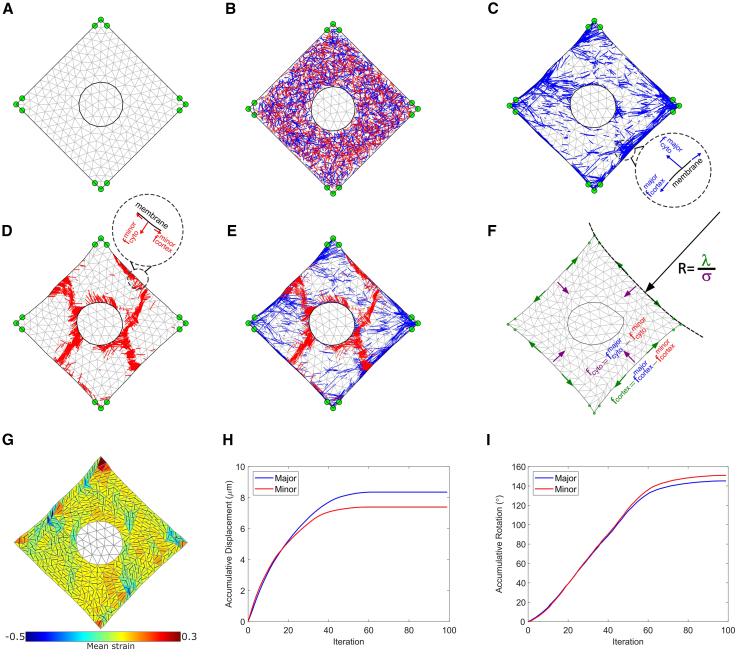
Model predictions (A) Similar to the finite element approach, the domain is discretized using a triangular mesh (B) Initial distribution of major and minor fibers. New arrangement of (C) major and (D) minor fibers after cell deformation. Insets show the force applied to a piece of plasma membrane by the cytoplasmic and cortical components of major and minor fibers. (E) Overlay of major and minor fibers. (F) Simple mechanical model and parameters of the contour model. (G) Distribution of mean strain within the domain, solid lines show the direction of the major principal strain in each element. (H) Accumulative average displacement and (I) rotation of major and minor fibers.

This section outlines the working principles of the model and its integration into the finite element framework. The initial step involves inputting the coordinates of both the cell’s peripheral points and the boundary points of the nucleus into the model. Subsequently, the domain is discretized employing a triangular mesh (Figure 2A), with major fibers (depicted in blue) and minor fibers (depicted in red), stochastically distributed across the domain, with a uniform distribution in both position and orientation (Figure 2B). The length of these fibers is randomly selected within specified maximum and minimum input values. Considering the Young’s modulus and Poisson’s ratio of the domain,^45^ the stiffness matrix is calculated for all elements. However, for elements containing a fiber, an additional term is added into the element’s stiffness matrix to incorporate the impact of the fiber (Equations S1–S4).

Following this, similar to a conventional finite element approach, all the stiffness matrices are assembled to construct the global stiffness matrix. At each iteration, unidimensional forces in the major fibers are computed using Equations S5–S10 as a function of current strain level in the fiber as suggested by Deshpande et al.^23^ These 1D forces are then interpolated using shape functions to find the equivalent nodal forces within elements containing the fibers (Equations S11 and S12). Subsequently, the nodal forces are assembled to generate the global nodal force vector *F*. Finally, the global equation *KU=F* is formed, where *K* is the global stiffness matrix, and U represents the nodal deformation vector. This global equation is solved after applying the boundary conditions at each iteration, and nodal displacements are calculated. The constraints at support points were modeled using springs in both x and y directions. Solving the system of equations, displacements and directional strains are computed at the center of each element with the aid of shape functions.

To determine the displacement of major fibers, a neighboring area around both ends of the fibers is searched to find a point with the highest mean principal strains. Based on the distance between this point and the filament’s end and the mean strain gradient, a dislocation vector is assigned to each end. The fiber displacement is the average of these dislocation vectors (Equation S13). Also, the directions of principal strains are found in the element containing the fiber’s midpoint. The fiber, then, rotates to align with the direction of the major principal strain (Equation S14). Similar rules are applied to minor fibers, but they move toward a point with the lowest mean strain and rotate to align with the direction of minor principal strain. Finally, a subroutine checks the new position of all fibers to ensure both ends are still located inside the cytoplasmic area. For the next iteration, the nodal force vector and stiffness matrix are updated.

A one-dimensional beam element was implemented around the cell and its nucleus to represent the roles of the plasma membrane and nuclear envelope, respectively. Additionally, the Young’s modulus and Poisson’s ratio of the nucleus can differ from those in the cytoplasmic region.

### Calculation of the radius of curvature

To quantify local curvature, both experimental and simulated images were first converted into binary masks. The cell boundary was detected from these masks and subsequently skeletonized using custom MATLAB scripts. Locations of FAs, obtained experimentally or defined in simulations, were used as reference points to partition the boundary into discrete segments. Each segment was then treated independently, and the local radius of curvature was calculated from the fitted arc to that segment using the least squares method (Methods S2). This approach allowed consistent measurement of curvature across multiple boundary regions, facilitating direct comparison between experimental data and numerical simulations.

### Quantification of the spatial distribution of fibers

To quantify the spatial distribution of actin and IFs within the cell, we computed a normalized distance from each fiber to the nucleus. First, the center of mass of the nucleus was calculated and denoted as point O (Figure S7). For each fiber, the coordinate of its geometric center was defined as point X. A line was drawn from O to X, and the intersections of this line with the nuclear boundary and the plasma membrane were identified as points A and B, respectively (Figure S7). The relative distance of the fiber from the nucleus was then calculated as the ratio AX/AB.

This dimensionless ratio provides a consistent spatial metric (ranging from 0 to 1) for comparing the localization of fibers within the cytoplasmic space, independent of cell size or shape. Further implementation details, quantification procedures, statistical analyses, and resource availability information are provided in the STAR Methods.

### Model predictions

#### Simulations in simplified geometry: Comparison with previous models

To better interpret the results of our full model and connect with existing theoretical frameworks, we first apply it to a simplified geometry - a square-shaped cell with a circular nucleus - and extract an idealized representation of the forces generated and tolerated by actin and IFs. This abstraction allows us to compare our results with previously developed simplified models,^21^^,^^46^ which we classify as conceptual or phenomenological models. While those models use analytically imposed force distributions or simplified tension fields to study cellular force organization, we extract the force patterns from finite element simulations based on biologically grounded filament rules. This comparison helps to isolate the roles of filament distribution and polarity in generating curvature and strain, demonstrating that key features such as localized deformation are preserved across modeling frameworks. It also strengthens the argument that spatial asymmetry and filament polarity are fundamental drivers of cell contraction, rather than artifacts of numerical implementation.

Model predictions demonstrate that major fibers exhibit similarities to the AFs, because they mainly accumulate near the FAs, cell cortex, and cytoplasmic region (Figure 2C). Closer examination of the major fibers in the vicinity of the plasma membrane reveals that while the cytoplasmic major fibers attempt to pull the membrane inside and increase the curvature (κ = 1/R), the cortex major fibers act in the other direction and try to decrease κ by stretching the membrane (Figure 2C).

Notably, minor fibers are predominantly located in the cytoplasmic region, while a small portion reside near the plasma membrane (Figure 2D). Investigation of the minor fibers positioned near the plasma membrane reveals that these 1D load-bearing elements increase the local stiffness and act against the excessive deformation of the plasma membrane (Figure 2D). The overlay of major and minor fibers is shown in Figure 2E.

To simplify the problem, we can calculate the resultant force originating from cortex and cytoplasmic major and minor fibers, this leads to a simplified mechanical model (Figure 2F). The distribution of the mean strains and the direction of major principal strains in each element at the last iteration is shown in Figure 2G. As can be seen, the mean strain shows local maxima near the four constraints, where the major fibers mainly accumulate. Also, the direction of major principal strains in the peripheral elements is typically parallel to the adjacent edges, which agrees with the orientation of AFs near the cortex.

It is worth mentioning that due to the complex nature of the problem and the interdependency of strain distribution and filaments' position, the final configuration of major and minor filaments is obtained through several iterations, allowing the smooth adjustments of the location and orientation of the filaments in each iteration. The simulation continues until both filaments reach a steady state (Figures 2H and 2I).

In our model, the radius of curvature (R), is controlled by the cortex and cytoplasmic component of resultant forces, which is consistent with a model previously developed by Bischofs et al.^21^ based on the Young-Laplace equation. In our model, the cortex and cytoplasmic force (f_cortex_ and f_cyto_) are equivalent to membrane tension (λ) and surface tension (σ) in the Bischofs’ model, respectively (Figure 2F). Furthermore, the mechanical system (Figure 2F) bears a striking resemblance to a previously proposed mechanical model developed by Tabdanov et al.,^46^ formulated based on experimental observations. Such similarities further validate the accuracy of our model. The dynamic evolution of major and minor fibers over time is presented in Video S1.

Video S1. Dynamic relocation and reorganization of AFs and IFs in the simplified cell geometry, related to Figure 2 The video shows the relocation and reorientation of actin filaments (AFs) and intermediate filaments (IFs) over 100 iterations for the model presented in Figure 2

To evaluate the mechanical role of IFs in cellular contractility, we performed a comparative simulation using the same setup as in Figure 2, but omitting IFs while retaining AFs, geometry, and boundary conditions (Figure S8). The results show that removing IFs increases both compressive and tensile mean strains (Figure S8A). Additionally, the extent of cellular deformation increases, resulting in a significant reduction in radius of curvature along the membrane (Figures S8B and S8C). These findings support the notion that IFs contribute to mechanical stability by resisting excessive deformation and regulating strain distribution.

To assess the robustness of the model with respect to stochastic initial conditions, we repeated the simulation shown in Figure 2 three times, each with a different randomly generated initial fiber distribution. All other parameters, geometry, and boundary conditions were kept identical, and each simulation was run for 100 iterations. The model consistently reproduced the final cell shape and curvature pattern across runs, with only minor differences arising from the randomness of the initial setup. In addition to membrane shape, the spatial distributions of actin and IFs also remained highly consistent across runs. Quantitative analysis of both membrane curvature and fiber distribution (Figure S9) confirms that the model yields reproducible and stable predictions despite stochastic variability in the initial conditions.

**Figure 3:**
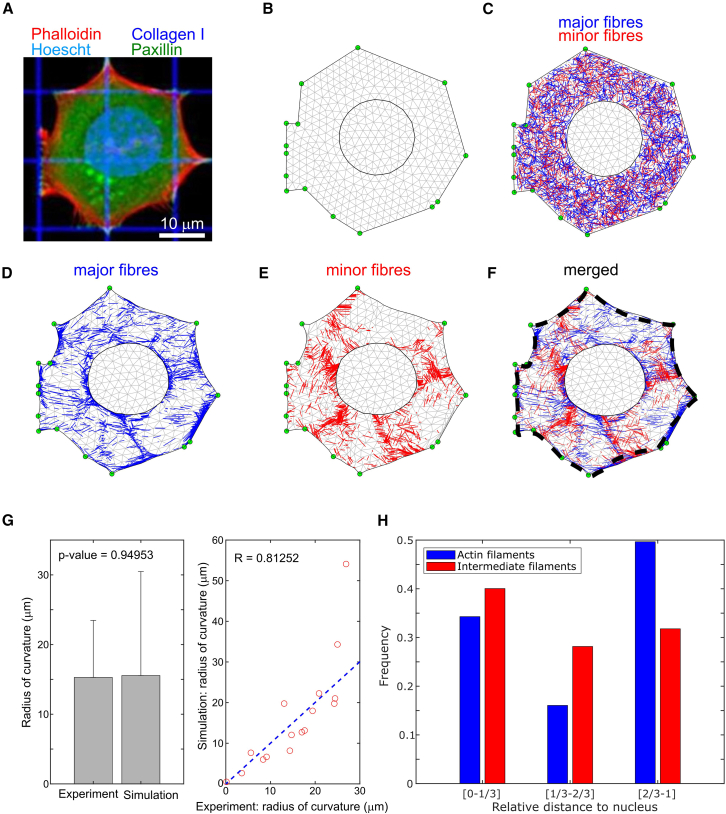
Comparison of model predictions with experimental data (A) Cells are cultured on a collagen I grid. F-actin was stained using Phalloidin and is shown in red, while paxillin labeling is shown in green (adapted from Tabdanov et al.^46^). (B) A triangular mesh was used to discretize the polygon representing cells. (C) Initial distribution of major and minor fibers over the undeformed domain. Focal adhesions are shown in green. (D) New arrangement of major and (E) minor fibers after cell deformation. (F) Overlay of major and minor fibers. Dash line depicts the experimental periphery of the cell. (G) Quantitative comparison of the radius of curvature of membrane segments in simulation and experimental data. *R* denotes the pairwise linear correlation coefficient. (H) Spatial distribution of actin and intermediate filaments within the cell, classified into three regions based on their relative distance from the nucleus: [0–1/3], [1/3–2/3], and [2/3–1]. (Upper error bars represent SD. *p* values were calculated using Student’s *t* test.).

**Figure 4:**
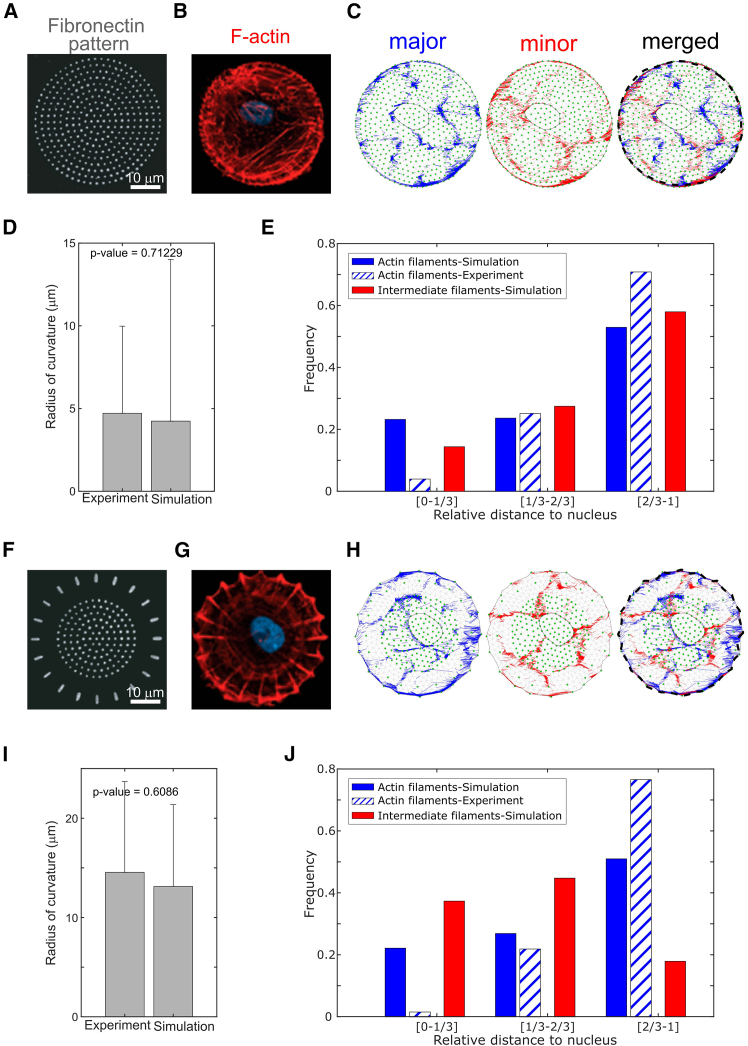
Comparison of model predictions with experimental data (A) Circular isotropic arrangement of fibronectin dots. (B) Filamentous actin in the cell cultured on the isotropic pattern. (C) Model’s prediction displaying the new arrangement of major and minor fibers after cell deformation. (D) Quantitative comparison of the radius of curvature of membrane segments in simulation and experimental data. (E) Spatial distribution of actin and intermediate filaments in the simulated and experimental cells. Filaments are grouped based on their relative distance to the nucleus. (F) Circular anisotropic arrangement of fibronectin dashes and dots. (G) Filamentous actin in the cell cultured on the anisotropic pattern. (H) Model’s prediction displaying the new arrangement of major and minor fibers after cell deformation. (I) Quantitative comparison of the radius of curvature of membrane segments in simulation and experimental data. (J) Spatial distribution of actin and intermediate filaments in the simulated and experimental cells. Filaments are grouped based on their relative distance to the nucleus. Dash line depicts the experimental periphery of the cell. Panels a, b, f, and g were adapted from Cabezas et al.^47^ (Upper error bars represent SD. *p* values were calculated using Student’s *t* test.).

**Figure 5:**
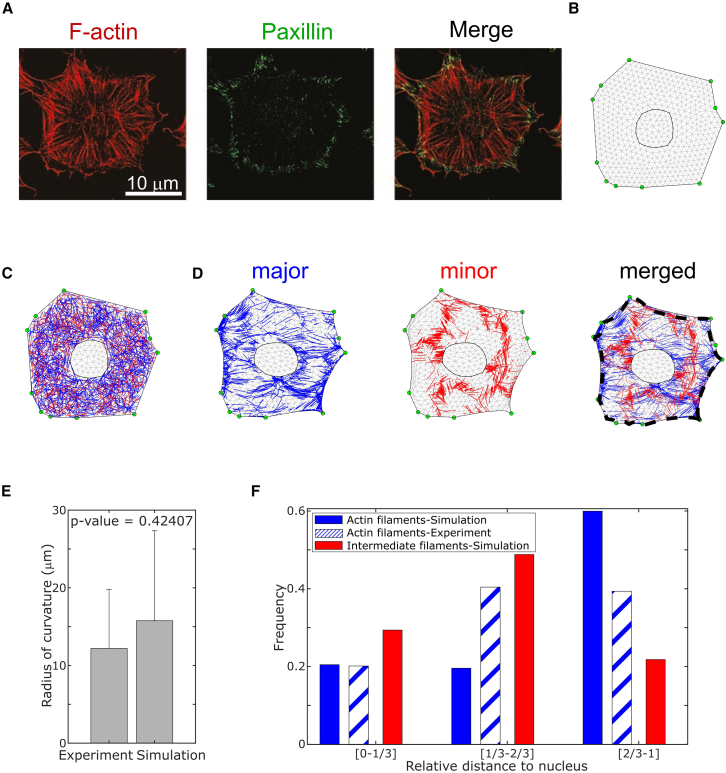
Comparison of model predictions with experimental data (A) NIH3T3cells, cultured on a fibronectin-coated plastic dish, were stained for F-actin and paxillin (Data adapted from Carter et al.^48^). (B) A polygon resembling the cell with a triangular mesh was used in the model. Constraints are depicted by green dots. (C) The initial distribution of major and minor fibers. (D) Predictions of the model show the accumulation of major fibers in the vicinity of the constraints and in the cytoplasmic region. Dash line depicts the experimental periphery of the cell. (E) Quantitative comparison of the radius of curvature of membrane segments in simulation and experimental data. (F) Spatial distribution of actin and intermediate filaments in the simulated and experimental cells. Filaments are grouped based on their relative distance to the nucleus. (Upper error bars represent SD. *p* values were calculated using Student’s *t* test.).

**Figure 6:**
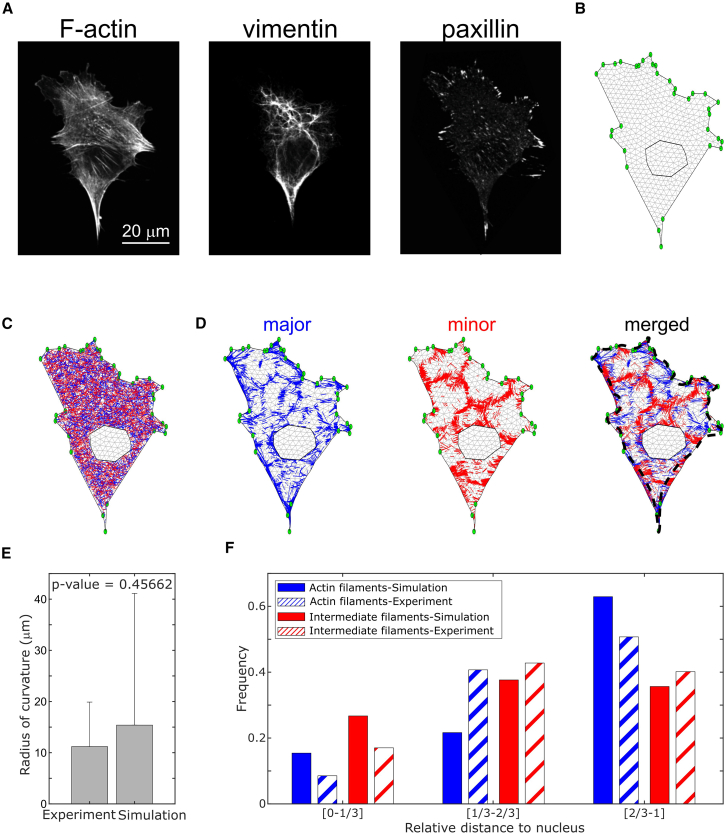
Comparison of model predictions with experimental data (A) Fibroblasts, cultured on glass, were stained for F-actin, vimentin, and paxillin (Data taken from Swoger et al.^49^). (B) A polygon resembling the cell with a triangular mesh was used in the model. Constraints are depicted by green dots. (C) The initial distribution of major and minor fibers. (D) Predictions of the model show the relative configuration of major and minor fibers within the cellular domain. Black dash-line represents the experimental periphery of the cell. (E) Quantitative comparison of the radius of curvature of membrane segments in simulation and experimental data. (F) Spatial distribution of actin and intermediate filaments in the simulated and experimental cells. Filaments are grouped based on their relative distance to the nucleus. (Upper error bars represent SD. *p* values were calculated using Student’s *t* test.).

To validate the model, we examine four scenarios and compare the model’s predictions with experimental data, focusing on the radius of curvature and the localization of major and minor fibers. In the first two conditions, a micropattern is printed on a surface to dictate the location of FAs (Figures 3 and 4), while in the other two cases, the cells are allowed to naturally form FAs by culturing them on a regular plastic or glass substrate (Figures 5 and 6).

### Cell on a substrate with predefined attachment sites

In this case, we use experimental data from Tabdanov et al.,^46^ where carcinoma cell line MDA-MB-468 was cultured on a polyacrylamide substrate functionalized with an orthogonally crossed pattern of collagen I (Figure 3A). The cells could adhere to the substrate only at their interface with collagen I.

To simulate cell contraction, a triangular mesh was used to discretize a polygon resembling the cell (Figure 3B) and the major and minor fibers were randomly distributed on the domain (Figure 3C). Video S2 shows how the major and minor fibers relocate and rotate to reach a steady state. Spatial distribution of major and minor fibers after 100 iterations is shown in Figures 3D and 3E.

Video S2. Dynamic relocation and reorganization of AFs and IFs for the collagen I grid condition, related to Figure 3 The video shows the relocation and reorientation of AFs and IFs over 100 iterations for the model presented in Figure 3

Quantitative comparison between experimental and simulated cell shapes demonstrates the model’s strong predictive capability in capturing local membrane curvature. The average radius of curvature in both experiment and simulation is approximately 15 μm, with a *p* value of 0.95 and a pairwise linear correlation coefficient of *R* = 0.81 (Figure 3G).

We also analyzed the spatial distribution of cytoskeletal filaments within the cell. In the simulations, ∼34% of AFs localized near the nucleus ([0–1/3]), ∼17% in the intermediate region ([1/3–2/3]), and ∼49% accumulated near the periphery ([2/3–1]), while IFs were more evenly distributed, with ∼40%, ∼28%, and ∼32% in the central, intermediate, and peripheral regions, respectively (Figure 3H). However, experimental data for IFs were not available, and due to cytoplasmic background staining, the precise spatial distribution of AFs across the whole cytoplasm could not be reliably quantified. As a result, no direct comparison between experimental and simulated fiber distributions was performed. Parameters used in the model for this case are listed in Table S1.

### Cell on a substrate with dash/dot-like attachment sites

In this case, we employ experimental data from Cabezas et al.,^47^ wherein human mesenchymal stem cells were cultured on a non-adherent substrate functionalized with an array of fibronectin dot/dash-like marks. Clearly, cells can only form FAs at the location of fibronectin marks.

To evaluate the efficacy of our model, we simulated two distinct examples and compared the results with experimental observations. In the first example, referred to as an isotropic condition, a circular pattern was formed using only dot-like marks (Figure 4A). The arrangement of filamentous actin (F-actin) is depicted in Figure 4B, and model predictions are presented in Figure 4C.

Quantitative comparison of local membrane curvature shows strong agreement, with the average radius of curvature measured as 4.2 μm in the experimental data and 3.8 μm in the simulation (*p* = 0.71; Figure 4D).

The spatial distribution of fibers further supports the model’s predictive capacity. In simulations, ∼15%, 28%, and 57% of IFs localize in the central, intermediate, and peripheral regions, respectively. For AFs, the simulated distributions are ∼23% (central), 23% (intermediate), and 54% (periphery). Experimental data also confirm that the majority of AFs (∼70%) accumulate near the cell periphery, while ∼5% and ∼23% are located near the nucleus and in the intermediate zone, respectively (Figure 4E). Although exact values differ, the model successfully captures the overall trend in AF distribution observed experimentally.

In the second example, referred to as an anisotropic condition, a circular configuration of FA sites was created using a combination of dashes and dots (Figure 4F). F-actin distribution and model predictions are displayed in Figures 4G and 4H, respectively.

Quantitative comparison of membrane curvature shows good agreement, with the average radius of curvature measured as 15 μm in the experiment and 13 μm in the simulation (*p* = 0.61; Figure 4I).

Fiber distribution analysis further supports the model’s ability to capture cytoskeletal organization. In simulations, IFs are distributed as ∼37%, 45%, and 18% in the central, intermediate, and peripheral zones, respectively. Simulated AFs show ∼22% near the nucleus, 27% in the intermediate region, and 51% near the periphery. Experimental data confirm a strong peripheral enrichment of AFs, with ∼77% located near the cell edge, 21% in the intermediate region, and only ∼2% near the nucleus (Figure 4J). These results demonstrate that the model successfully replicates the overall spatial trend in actin distribution under anisotropic conditions.

It is worth noting that both models employ identical parameters (Table S1). Therefore, we can infer that the model is capable of predicting cell contraction in scenarios involving both isotropic and anisotropic FAs. The dynamic relocation and orientation of the fibers for the first and second scenarios are shown in Videos S3 and S4, respectively.

Video S3. Dynamic relocation and reorganization of AFs and IFs for the isotropic adhesion pattern, related to Figure 4 The video shows the relocation and reorientation of AFs and IFs over 150 iterations for the model presented in Figures 4A–4E

Video S4. Dynamic relocation and reorganization of AFs and IFs for the anisotropic adhesion pattern, related to Figure 4 The video shows the relocation and reorientation of AFs and IFs over 150 iterations for the model presented in Figures 4F–4J

### Configuration of filaments in a 2D culture of cells

In this case, we utilized experimental data from Carter et al.,^48^ where NIH3T3cells were cultured on a fibronectin-coated plastic dish. Therefore, cells were allowed to naturally form their FAs on a 2D surface. Phalloidin was used to detect the F-actin and to visualize FAs, the cells were stained for paxillin using antibodies (Figure 5A).

To assess the efficiency of the model, we initially discretized a polygon resembling the cell using a triangular mesh (Figure 5B). The location of the constraints, indicated by green dots in Figure 5B, was selected based on the localization of paxillin in Figure 5A. The initial distribution of major and minor fibers over the domain is shown in Figure 5C, while predictions of the model are presented in Figures 5D and Video S5. Parameters used in the model for this case are listed in Table S1.

Video S5. Dynamic relocation and reorganization of AFs and IFs for the NIH3T3cell model, related to Figure 5 The video shows the relocation and reorientation of AFs and IFs over 150 iterations for the model presented in Figure 5

Quantitative comparison of local membrane curvature reveals close agreement, with an average radius of curvature of 13 μm in the experimental data and 16 μm in the simulation (*p* = 0.42; Figure 5E).

Fiber distribution analysis shows that simulated IFs are distributed as ∼29% in the central region, 49% in the intermediate zone, and 22% near the periphery. Simulated AFs show stronger peripheral localization, with ∼20%, 20%, and 60% in the central, intermediate, and peripheral regions, respectively. Experimental measurements of AFs indicate ∼20% near the nucleus, 40% in the intermediate region, and 40% at the periphery (Figure 5F). These results confirm the model’s ability to approximate the general spatial organization of AFs and capture curvature features consistent with experimental observations.

### Configuration of filaments in a 2D culture of fibroblast

In this case, we used experimental data from Swoger et al.,^49^ where the mouse embryonic fibroblast was cultured on a collagen-coated glass slide, allowing the cells to naturally develop their FAs. The cells were stained for F-actin, paxillin, and vimentin, one of the most abundant members of the IF family,^50^ enabling us to visualize the relative location of AFs and IFs (Figure 6A).

We employed a polygon resembling the cell to simulate cellular behavior (Figure 6B), over which major and minor fibers were initially distributed randomly (Figure 6C). The location of the constraints is indicated by green dots in Figures 6B–6D, was selected based on the localization of paxillin in Figure 6A. Model predictions are presented in Figure 6D and Video S6. Parameters used in the model for this case are listed in Table S1.

Video S6. Dynamic relocation and reorganization of AFs and IFs for the fibroblast model, related to Figure 6 The video shows the relocation and reorientation of AFs and IFs over 150 iterations for the model presented in Figure 6

Quantitative comparison of curvature shows good agreement between model and experiment, with average radius of curvature measured as 12 μm and 16 μm in the experimental and simulated data, respectively (*p* = 0.46; Figure 6E).

Fiber distribution analysis also reveals strong alignment between predictions and observations. Simulated AFs are distributed as ∼15%, 21%, and 64% in the central, intermediate, and peripheral regions, respectively, while experimental data show ∼9%, 40%, and 51% in the same zones (Figure 6F). For IFs, the simulation predicts ∼26% (central), 38% (intermediate), and 36% (periphery), while the corresponding experimental measurements are ∼18%, 42%, and 40%. These results indicate that the model effectively captures both global morphological features and the general trends in filament organization.

## Discussion

In this study, we focused on cell contraction, a fundamental process involved in many biological processes. Our model offers a continuous-discrete approach for predicting cytoplasmic filament dynamics by incorporating the distinct roles of AFs and IFs in cell contractility. It leverages a finite element framework where randomly distributed filaments can relocate and reorient based on the spatial distribution of the strain tensor. Our model considers the gradient of mean principal strain as a guiding factor for filament movement within the cell. Additionally, the assumption that filaments tend to align with major and minor principal strains leads to realistic predictions of filament orientation, further validating our approach. Furthermore, by incorporating one-dimensional elements and a state-dependent law for active force generation by AFs, our model captures the radius of curvature of the plasma membrane, providing a more comprehensive representation of cellular mechanics.

By applying our model to a reduced geometric setting and comparing the resulting force patterns with simplified theoretical frameworks, we demonstrate that the fundamental mechanical principles underlying filament-mediated contraction are preserved across levels of abstraction. This comparison not only supports the validity of our full model but also enhances interpretability by linking it to earlier conceptual approaches. Simplified comparisons such as these serve as valuable tools for testing, validating, and explaining complex computational models.

It is worth noting that the code was developed using a modular design and validated through unit testing, where individual functions are tested separately to confirm that they produce the expected outputs, and *in silico* perturbation experiments to ensure the model components function as intended. All functions were tested independently, and the system behavior was benchmarked under simplified scenarios to confirm model fidelity.

In agreement with experimental observations, our *in silico* experiments demonstrated that major fibers, akin to AFs, aggregated near FAs, the plasma membrane, or formed bundles within cytoplasm. Interestingly, FAs' configuration significantly influenced strain distribution and, consequently, final AFs positioning. In our model, spring elements representing FAs restricted nodal displacements. By adjusting spring stiffness, we observed that stiffer constraints attracted more major fibers. Minor fibers, mimicking IFs, prevented excessive deformation by absorbing forces and increasing local stiffness. Notably, regions with high concentrations of minor fibers near the plasma membrane (Figures 4B and 4C) exhibit negligible curvature. Conversely, when the minor fibers are more dispersed throughout the cytoplasm (Figures 4G and 4H), the membrane’s curvature is considerably greater.

In conclusion, this model offers a useful framework to investigate the interplay between AFs, IFs, FAs, and strain distribution in regulating cell contraction. By capturing the dynamic behavior of these elements, the model provides valuable insights into the mechanisms underlying this fundamental cellular process.

### Limitations of the study

The current model has several limitations. First, it does not explicitly include the effects of external forces or the mechanical properties of the extracellular matrix (ECM) on filament dynamics and contractility regulation. Second, the polymerization and dissociation of AFs and IFs are not simulated explicitly; instead, filament relocation and reorientation are implemented through imposed strain-based rules. Third, the model does not include interactions with neighboring cells, which may be important in multicellular environments. Finally, the model is currently formulated in two dimensions, whereas a 3D framework would provide a more realistic representation of cellular behavior in physiological environments.

## Resource availability

### Lead contact

Further information and requests for resources should be directed to and will be fulfilled by the lead contact, Fabian Spill (f.spill@bham.ac.uk).

### Materials availability

This study did not generate new unique reagents.

### Data and code availability

• Data: This study did not generate new biological datasets requiring deposition in a community-endorsed repository. Experimental images and data used for model validation were obtained from previously published studies, as cited in the relevant figure legends and key resources table.
• Code: The code generated for this study has been deposited on Zenodo: https://doi.org/10.5281/zenodo.19967673, and is also available on GitHub at https://github.com/yousefjavanmardi/Cell_Contractility.git.
• Other items: Any additional information required to reanalyze the data reported in this paper is available from the lead contact upon request.

## Acknowledgments

E.M. is grateful for the Wellcome Trust-MIT Fellowship (WT103883). Y.J. and E.M. acknowledge financial support by Leverhulme Trust Research Project Grant (RPG-2018-443) and the Cancer Research UK Multidisciplinary Award (C57744/A22057). Y.J., E.M., and F.S acknowledge support from the 10.13039/501100000268Biotechnology and Biological Sciences Research Council Grant (BB/V001418/1). F.S. was supported by a UKRI Future Leaders Fellowship, grant number MR/T043571/1.

## Author contributions

Conceptualization: S.S. and Y.J.; methodology: S.S. and Y.J.; software: S.S., Y.J., R.A., and M.N.; formal analysis: S.S. and Y.J.; investigation: S.S., Y.J., R.A., and M.N.; validation: S.S. and Y.J.; visualization: S.S. and Y.J.; writing – original draft: S.S., Y.J., R.A., M.N., and P.K.; writing – review and editing: S.S., Y.J., P.K., E.M., and F.S.; supervision: E.M. and F.S.; funding acquisition: E.M. and F.S.

## Declaration of interests

The authors declare no competing interests.

## STAR★Methods

### Key resources table

**Table:** 

REAGENT or RESOURCE	SOURCE	IDENTIFIER
**Deposited data**		

Cell_Contractility code	This paper; Zenodo	https://doi.org/10.5281/zenodo.19967673
Cell_Contractility GitHub repository	This paper; GitHub	https://github.com/yousefjavanmardi/Cell_Contractility.git

**Software and algorithms**		

MATLAB R2024a	MathWorks	https://www.mathworks.com/products/matlab.html

**Other**		

MDA-MB-468 collagen I grid image	Tabdanov et al.^46^	N/A
hMSC fibronectin dot/dash pattern images	Cabezas et al.^47^	N/A
NIH3T3 F-actin/paxillin image	Carter et al.^48^	N/A
Fibroblast F-actin/vimentin/paxillin image	Swoger et al.^49^	N/A

### Experimental model and study participant details

Omitted as this study does not involve new biological models, human participants, animals, primary cell cultures, cell lines, plants, or microbe strains. Experimental images and measurements used for model validation were obtained from previously published studies and are cited in the relevant figure legends and key resources table. Therefore, no new ethical approval, cell-line authentication, mycoplasma testing, maintenance/care information, or sex/gender-based analysis was required for this study.

### Method details

#### Model overview

The model was developed as a two-dimensional continuous-discrete finite element framework to simulate cell contraction. The cytoplasmic domain and nucleus were discretised using triangular finite elements, while actin filaments (AFs) and intermediate filaments (IFs) were represented as one-dimensional elements embedded within the cellular domain. AFs generated active contractile forces, whereas IFs contributed to local mechanical stiffness and resistance to excessive deformation.

#### Geometry and mesh generation

Cell and nuclear boundaries were defined from experimental images or representative polygonal geometries. The cellular domain was discretised using a triangular mesh. A one-dimensional beam element was implemented around the cell and nucleus to represent the plasma membrane and nuclear envelope, respectively. Focal adhesions or attachment sites were represented as spring constraints applied at selected boundary locations.

#### Filament initialization

AFs and IFs were initially distributed randomly within the cytoplasmic domain. Their lengths, positions, and orientations were randomly assigned within prescribed ranges. The initial filament distribution was then updated iteratively during cell deformation according to strain-based relocation and reorientation rules.

#### Force generation and finite element solution

AFs generated active contractile dipole forces based on the state-dependent formulation described in the manuscript and Methods S1. These forces were interpolated to the finite element nodes using shape functions. The global finite element equation was assembled and solved at each iteration after applying the relevant boundary conditions. Elements containing embedded filaments included an additional stiffness contribution from the one-dimensional filament element.

#### Filament relocation and reorientation

At each iteration, local strain fields were calculated throughout the cytoplasmic domain. AFs were moved toward neighboring regions of maximum mean principal strain and reoriented toward the major principal strain direction. IFs were moved toward neighboring regions of minimum mean principal strain and reoriented toward the minor principal strain direction. Filament positions were checked after each iteration to ensure that both endpoints remained inside the cytoplasmic domain.

#### Curvature analysis

Experimental and simulated cell boundaries were converted into binary masks and skeletonised using custom MATLAB scripts. Boundary segments were defined using focal adhesion locations or attachment points as references. The local radius of curvature of each segment was calculated by fitting a circle to the corresponding boundary section using a least-squares method, as described in Methods S2.

#### Fiber distribution analysis

The spatial distribution of AFs and IFs was quantified using a normalised distance from the nucleus. For each filament, the geometric center was identified, and its relative position between the nuclear boundary and plasma membrane was calculated. Filaments were classified into three regions based on their relative distance from the nucleus: [0–1/3], [1/3–2/3], and [2/3–1].

#### Published experimental data

No new experimental data were generated in this study. Experimental images and measurements used for validation were obtained from previously published studies and are cited in the relevant figure legends. Therefore, no new ethical approval was required for this study.

### Quantification and statistical analysis

Statistical analyses were performed as indicated in the relevant figure legends. Student’s *t* test was used for comparisons in Figures 3, 4, 5, 6, and S8. One-way ANOVA was used for the reproducibility analysis in Figure S9 *p*-values are reported directly in the figures or figure legends. Statistical significance is indicated only in Figure S8, where ∗*p* < 0.05. Upper error bars represent SD where shown. Statistical analyses and plotting were performed using MATLAB R2024a.
