# Supplementary material for: A continuous-discrete model of cell contraction incorporating actin and intermediate filaments
Source: iScience. 2026 Jun 5;29(6):116279. doi: 10.1016/j.isci.2026.116279 (PMC13264378; doi:10.1016/j.isci.2026.116279)
Supplement: Document S1. Figures S1–S9, Table S1, Methods S1 and S2 [file mmc1.pdf]

iScience, Volume *29*

Supplemental information

A continuous-discrete model of cell contraction incorporating actin and intermediate filaments

Soheil Sarbishei, Yousef Javanmardi, Reza Azarbad, Morteza Naeij, Pradeep Keshavanarayana, Emad Moeendarbary, and Fabian Spill

Methods S1. Composite element stiffness matrix and fibre dynamics, related to STAR Methods.

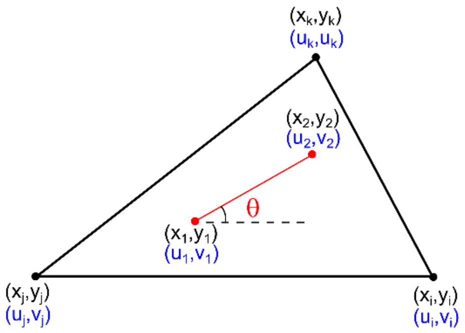

Supplementary Figure 1. Schematic composite element (triangular cell element with one dimensional filament element)

The stiffness matrix for a typical element shown **in Supplementary Fig. 1** is calculated using **Supplementary Eq. 1-4**:

$$Potential\ Energy = \int [U_F]^t [K_F][U_F] + \int [U_{cell}]^t [K_{cell}][U_{cell}] \quad (1)$$

$$[U_{SF}] = \begin{bmatrix} u_1 \\ v_1 \\ u_2 \\ v_2 \end{bmatrix} \qquad [U_{cell}] = \begin{bmatrix} u_i \\ v_i \\ u_j \\ v_j \\ u_k \\ v_k \end{bmatrix} \quad (2)$$

$$\begin{bmatrix} u_1 \\ v_1 \\ u_2 \\ v_2 \end{bmatrix} = \underbrace{\begin{bmatrix} N_i(x_1,y_1) & 0 & N_j(x_1,y_1) & 0 & N_k(x_1,y_1) & 0 \\ 0 & N_i(x_1,y_1) & 0 & N_j(x_1,y_1) & 0 & N_k(x_1,y_1) \\ N_i(x_2,y_2) & 0 & N_j(x_2,y_2) & 0 & N_k(x_2,y_2) & 0 \\ 0 & N_i(x_2,y_2) & 0 & N_j(x_2,y_2) & 0 & N_k(x_2,y_2) \end{bmatrix}}_{[\bar{N}]} \begin{bmatrix} u_i \\ v_i \\ u_j \\ v_j \\ u_k \\ v_k \end{bmatrix} \quad (3)$$

$$[U_{SF}] = [\bar{N}][U_{cell}]$$

$$Potential\ Energy = \int [U_{cell}]^t [\bar{N}]^t [K_F][\bar{N}][U_{cell}] + \int [U_{cell}]^t [K_{cell}][U_{cell}] \quad (4)$$

$$[K_{composite}] = [\bar{N}]^t [K_F][\bar{N}] + [K_{cell}]$$

In the above equations K_F and K_{cell} are the stiffness matrix of the filament and cell, respectively.

Applying Stress Fibre Force

The evolution of the activation level of actin fibres (AFs) is computed based on the work of Deshpande et al. (1) following the below equations:

For each fibre, the strain rate at time step $t + \Delta t$ is calculated using **Supplementary Eq. 5**:

$$\varepsilon^{\cdot t+\Delta t} = \frac{\frac{L^{t+\Delta t} - L^{t}}{L^{t}}}{\Delta t} \tag{5}$$

where $\varepsilon^{\cdot t+\Delta t}$ is the axial compressive strain, L is the length of the fibre, and Δt is the time increment. Superscripts t and $t+\Delta t$ denote the time steps. Next, the fibre lengthening is calculated using **Supplementary Eq. 6**:

$$\frac{\sigma}{\sigma_0} = \begin{cases} 0 & \frac{\dot{\varepsilon}}{\dot{\varepsilon_0}} \leq -\frac{\eta}{k_v} \\ 1 + \frac{k_v}{\eta}\frac{\dot{\varepsilon}}{\dot{\varepsilon_0}} & -\frac{\eta}{k_v} < \frac{\dot{\varepsilon}}{\dot{\varepsilon_0}} \leq 0 \\ 1 & 0 < -\frac{\dot{\varepsilon}}{\dot{\varepsilon_0}} \end{cases} \tag{6}$$

in which σ is the stress in the fibre, k_v and $\dot{\varepsilon_0}$ are model parameters, and σ_0 is the isometric stress at activation level η which is computed using **Supplementary Eq. 7**:

$$\sigma_0 = \eta.\sigma_{max} \tag{7}$$

where σ_{max} is the tensile stress that the stress fibres exert at full activation ($\eta = 1$). $\sigma_{max}$is a model parameter. The activation level η is calculated using **Supplementary Eq. 8-10**:

$$\dot{\eta} = (1-\eta)\frac{C\bar{k}_f}{\theta} - \left(1 - \frac{\sigma(\varphi)}{\sigma_0}\right)\eta\frac{\bar{k}_b}{\theta} \tag{8}$$

$$C = C_{signal}exp\left(\frac{-t}{\theta}\right) \tag{9}$$

$$\eta^{t+\Delta t} = \eta^{t} + \dot{\eta}.\Delta t \tag{10}$$

where C represents the signal level, specifically denoting the concentration of Ca^{2+} ions, constrained within the range of 0≤C≤1. θ is a constant representing the decay of the signal. In the absence of any external signal C_{signal} is assumed to be 0, otherwise $C_{signal} = 1$. t is the time measured from the instant of most recent signal. The over-dot notation denotes the rate of change of h over time. Dimensionless constants $\bar{k}_f$ and $\bar{k}_b$ define formation and dissociation of the AFs, respectively.

It's worth noting that a planar 3-node triangular element has six degree of freedom (two at each node); the displacements at the ends of the filaments are calculated from nodal displacements using shape functions. Furthermore, the concentrated force in the filament is translated to the nodal force using the shape functions, therefore, addition of the one-dimensional elements, do not add any further degree of freedom to the model.

Applying active nodal force

Two scenarios are considered to determine the equivalent nodal forces:

i) When the entire AF lies within a single triangular element, the nodal forces can be directly computed based on the shape functions associated with that specific element (**Supplementary Fig. 2a** and **Supplementary Eq. 11**).

ii) When the AF extends across multiple triangular elements (**Supplementary Fig. 3** and **Supplementary Eq. 12**).

To provide a visual understanding, schematic representations of the equivalent nodal forces resulting from the AFs' dipole forces are depicted in **Supplementary Fig. 2** and **3**.

For a more comprehensive understanding of this process, the following equations provide detailed insight.

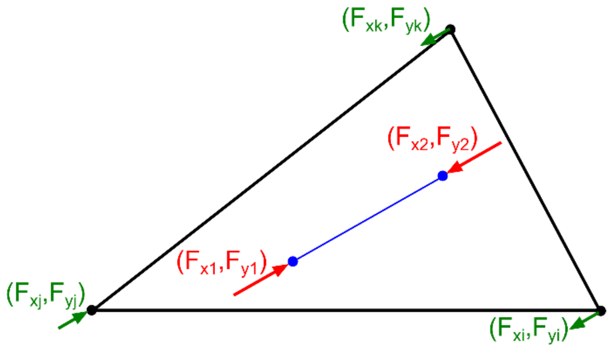

Supplementary Figure 2. Equivalent nodal forces when entire AF lies within single triangular element.

$$\begin{bmatrix} F_{x_i} \\ F_{y_i} \\ F_{x_j} \\ F_{y_j} \\ F_{x_k} \\ F_{y_k} \end{bmatrix} = \begin{bmatrix} F_{x_1}\left(N_i(x_1,y_1) - N_i(x_2,y_2)\right) \\ F_{y_1}\left(N_i(x_1,y_1) - N_i(x_2,y_2)\right) \\ F_{x_1}\left(N_j(x_1,y_1) - N_j(x_2,y_2)\right) \\ F_{y_1}\left(N_j(x_1,y_1) - N_j(x_2,y_2)\right) \\ F_{x_1}\left(N_k(x_1,y_1) - N_k(x_2,y_2)\right) \\ F_{y_1}\left(N_k(x_1,y_1) - N_k(x_2,y_2)\right) \end{bmatrix} \tag{11}$$

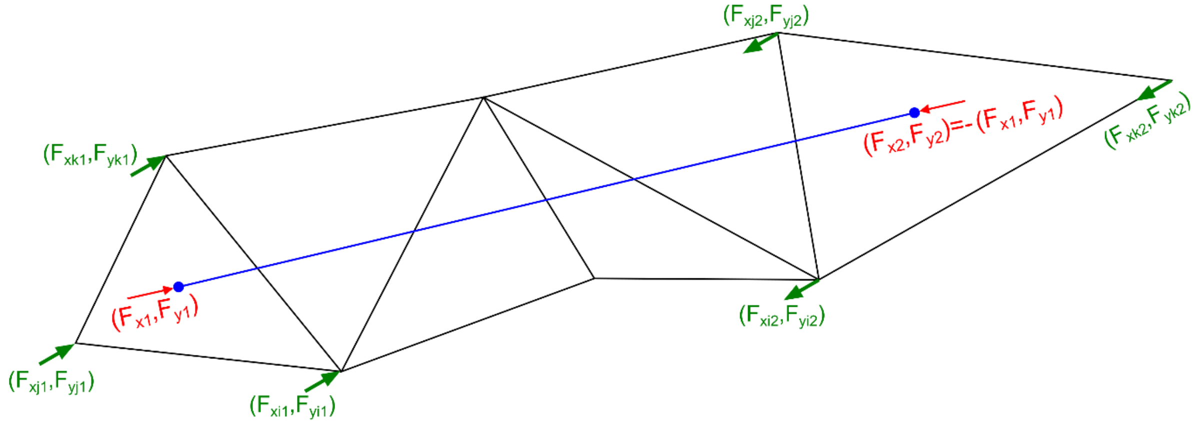

Supplementary Figure 3. Equivalent nodal forces when AF extended across multiple triangular elements.

$$\begin{bmatrix} F_{x_{i1}} \\ F_{y_{i1}} \\ F_{x_{j1}} \\ F_{y_{j1}} \\ F_{x_{k1}} \\ F_{y_{k1}} \end{bmatrix} = \begin{bmatrix} F_{x_1} N_i(x_1, y_1) \\ F_{y_1} N_i(x_1, y_1) \\ F_{x_1} N_j(x_1, y_1) \\ F_{y_1} N_j(x_1, y_1) \\ F_{x_1} N_k(x_1, y_1) \\ F_{y_1} N_k(x_1, y_1) \end{bmatrix} \qquad \begin{bmatrix} F_{x_{i2}} \\ F_{y_{i2}} \\ F_{x_{j2}} \\ F_{y_{j2}} \\ F_{x_{k2}} \\ F_{y_{k2}} \end{bmatrix} = \begin{bmatrix} F_2 N_i(x_2, y_2) \\ F_{y_2} N_i(x_2, y_2) \\ F_{x_2} N_j(x_2, y_2) \\ F_{y_2} N_j(x_2, y_2) \\ F_{x_2} N_k(x_2, y_2) \\ F_{y_2} N_k(x_2, y_2) \end{bmatrix} = - \begin{bmatrix} F_{x_1} N_i(x_2, y_2) \\ F_{y_1} N_i(x_2, y_2) \\ F_{x_1} N_j(x_2, y_2) \\ F_{y_1} N_j(x_2, y_2) \\ F_{x_1} N_k(x_2, y_2) \\ F_{y_1} N_k(x_2, y_2) \end{bmatrix} \quad (12)$$

Fibre dynamics

To obtain the relocation of the actin fibre AB (**Supplementary Fig. 4**), a region around both ends A and B is searched (red circles in **Supplementary Fig. 4**). Mean principal strain is calculated at the centre of all elements located inside the search area (at green dots in **Supplementary Fig. 4**) and the maximum and minimum values are found (corresponding to the black and orange dots in **Supplementary Fig. 4**). Then, a dislocation vector is calculated at both points A and B using **Supplementary Eq. 13**:

$$\Delta u_A = DF.d.\frac{\bar{\varepsilon}_{max} - \bar{\varepsilon}_A}{RangeE}$$

$$\Delta u_B = DF.d.\frac{\bar{\varepsilon}_{max} - \bar{\varepsilon}_B}{RangeE} \quad (13)$$

where Δu_A and Δu_B are the dislocation vectors at A and B, DF is a model parameter, controlling how fast the fibres move, d is the Euclidian distance between A (or B) and the point with maximum mean strain at which strain is $\bar{\varepsilon}_{max}$, $\bar{\varepsilon}_A$ is the mean strain at A, and $RangeE$ is calculated by subtracting the minimum mean strain from the maximum mean strain in whole cytoplasmic area. Finally, the displacement vector $\Delta u = (\Delta u_A + \Delta u_B)/2$ is applied to the fibre. The same procedure is followed to find the dislocation vector for the intermediate filaments (IFs), the only difference is that $\bar{\varepsilon}_{max}$ is replaced with $\bar{\varepsilon}_{min}$ (**Supplementary Fig. 4**).

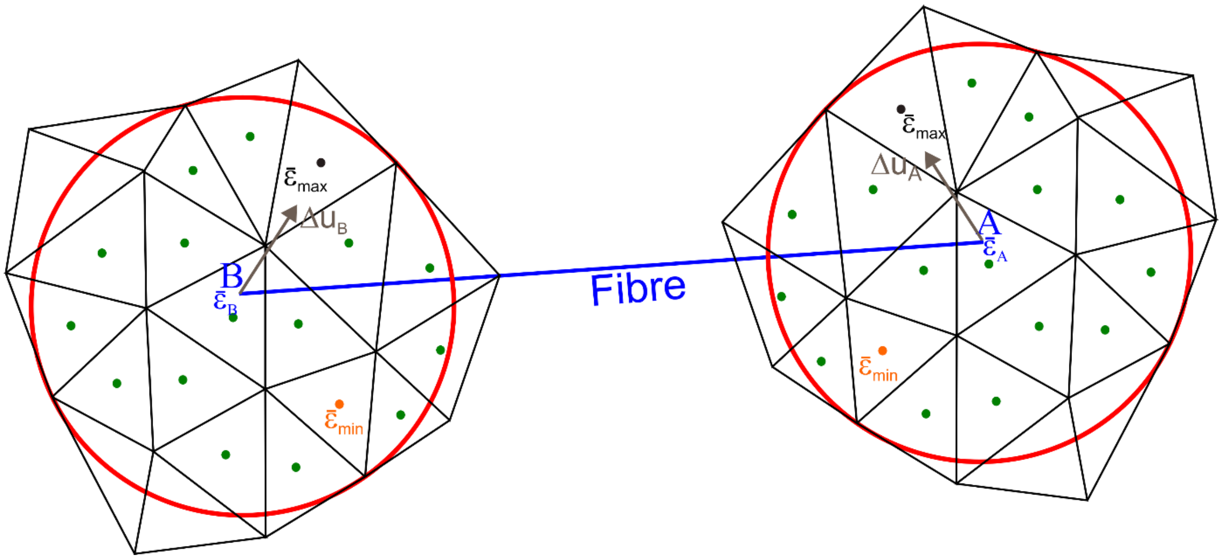

Supplementary Figure 4. Determining displacement vector of fibres.

To calculate the rotation of each actin filament, the direction of major principal strain is found at the centre of the element containing midpoint of the fibre (**Supplementary Fig.5**). The angle between the fibre and vertical axis is shown by θ and the one between the direction of major principal strain and the vertical axis is denoted by φ. The rotation of the actin filament is determined by **Supplementary Eq. 14**:

$$\Delta\theta = RF.\frac{\varphi - \theta}{Range\theta} \tag{14}$$

where $\Delta\theta$ is the rotation angle of the fibre, RF is a model parameter, controlling how fast the fibres rotates, and $Range\theta$ is calculated by subtracting the minimum value of φ from its maximum value throughout the cytoplasmic area. The same procedure is followed to find the rotation angle for the IFs, the only difference is that φ denoted the angle between minor principal strain and vertical axis (**Supplementary Fig. 5**).

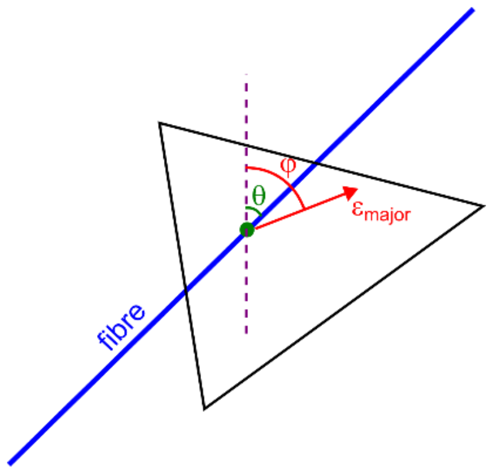

Supplementary Figure 5. Determining reorientation of fibres.

In summary, at each iteration, the following steps are performed:

1. Compute strain and principal directions for each element (**Supplementary Eq. 5-12**).
2. For each filament:

    a. Find the strain extrema (max or min) in the vicinity of its ends.

    b. Apply displacement based on the gradient and distance to the extremum (**Supplementary Eq. 13**).

    c. Rotate towards principal strain direction (**Supplementary Eq.14**).

3. Ensure all filaments remain inside the cytoplasm.
4. Update global stiffness matrix and solve for displacements (**Supplementary Eq. 1-4**).
5. Repeat until convergence is achieved.

Values of Parameters in Models

In all cases, parameters in Supplementary Eq. 6-9 are selected as below: $\theta = 300$, $k_f = 10$, $k_b = 1$, $\dot{\varepsilon}_0 = 0.0028$, $k_v = 10$, and $C_{signal} = 0$ (all taken from Deshpande et al. [S1]). Poisson's ratio for both cytoplasm and nucleus is 0.3. The remaining parameters utilised in analyses are listed in **Supplementary Table 1**. Young's modulus of cells was taken from Barzegari et al. [S2].

Supplementary Table 1. Parameters utilised in analyses.

Parameters	Description	Unit	Value (Fig. 2)	Value (Fig. 3)	Value (Fig. 4)	Value (Fig. 5)	Value (Fig. 6)
E_{cell}	Cell Young's modulus	kPa	0.5	0.5	10	0.4	2
$E_{Nucleus}$	Nuclear Young's modulus	kPa	1.5	2	20	0.8	4
σ_{max}	AFs elasticity modulus	kPa	2.5	7	3	4	9
DF_{major}	Displacement factor-major fibres	--	0.2	0.1	0.06	0.15	0.25
RF_{major}	Rotation factor-major fibres	--	0.2	0.2	0.15	0.2	0.2
DF_{minor}	Displacement factor-minor fibres	--	0.2	0.1	0.07	0.15	0.25
RF_{minor}	Rotation factor-minor fibres	--	0.2	0.2	0.08	0.2	0.2
$K_{support}$	Spring constant	pN/mm	20000	20000	5000-20000	20000	20000

Regarding the spring constants, in **Fig. 4a-c** $K_{support} = 5000\, pN/\mu m$ for all supports. In **Fig. 4d-f**, $K_{support} = 5000\, pN/\mu m$ for the supports located in the central region of cell and $K_{support} = 20000\, pN/\mu m$ for the supports located near the plasma membrane. Here, to incorporate the effect of larger FAs, a higher spring constant was adopted for the outermost supports.

Methods S2. Least-squares estimation of radius of curvature, related to STAR Methods.

The local radius of curvature for each boundary segment was obtained by fitting a circle using a least-squares approach. The general equation of a circle is:

$$(x-a)^2 + (y-b)^2 = R^2 \tag{15}$$

which can be rewritten in linearised form as:

$$x^2 + y^2 + A\,x + B\,y + C = 0 \tag{16}$$

where $A = -2a$, $B = -2b$, and $C = a^2 + b^2 - R^2$.

For a set of nodes (x_i, y_i) belonging to a given boundary segment, we minimise the error function $S = \sum_i \left(x_i^2 + y_i^2 + A\,x_i + B\,y_i + C\right)^2$. Setting the partial derivatives of S with respect to A, B, and C equal to zero yields the following linear system of equations:

$$\begin{bmatrix} \sum_i x_i^2 & \sum_i x_i y_i & \sum_i x_i \\ \sum_i x_i y_i & \sum_i y_i^2 & \sum_i y_i \\ \sum_i x_i & \sum_i y_i & n \end{bmatrix} \begin{Bmatrix} A \\ B \\ C \end{Bmatrix} = \begin{Bmatrix} -\sum_i x_i z_i \\ -\sum_i y_i z_i \\ -\sum_i z_i \end{Bmatrix} \tag{17}$$

where $z_i = x_i^2 + y_i^2$ and n is the number of nodes.

Solving this system gives the coefficients A, B, and C, from which the circle center (a, b) and radius R are obtained as: $a = -A/2$, $b = -B/2$, and $R = \sqrt{a^2 + b^2 - C}$.

This procedure was applied independently to each boundary section defined between two adjacent focal adhesion points (**Supplementary Fig. 6**).

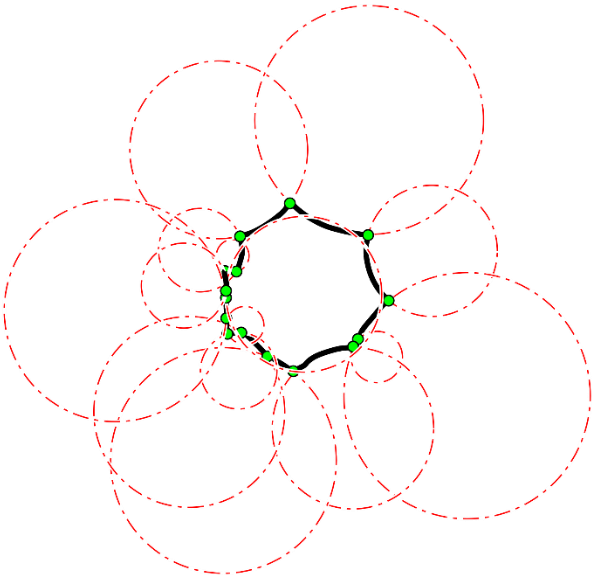

Supplementary Figure 6. Determination of radius of curvature for membrane sections using circle fitting

Quantification of Spatial Distribution of Fibres

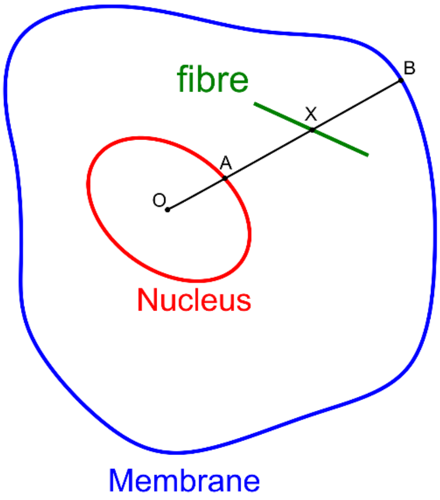

Supplementary Figure 7. Determination of the relative distance to the nucleus for each fibre. The nucleus centre is labelled as O, the fibre center as X, and the intersection points with the nuclear and cell boundaries as A and B, respectively. The relative distance is calculated as AX/AB.

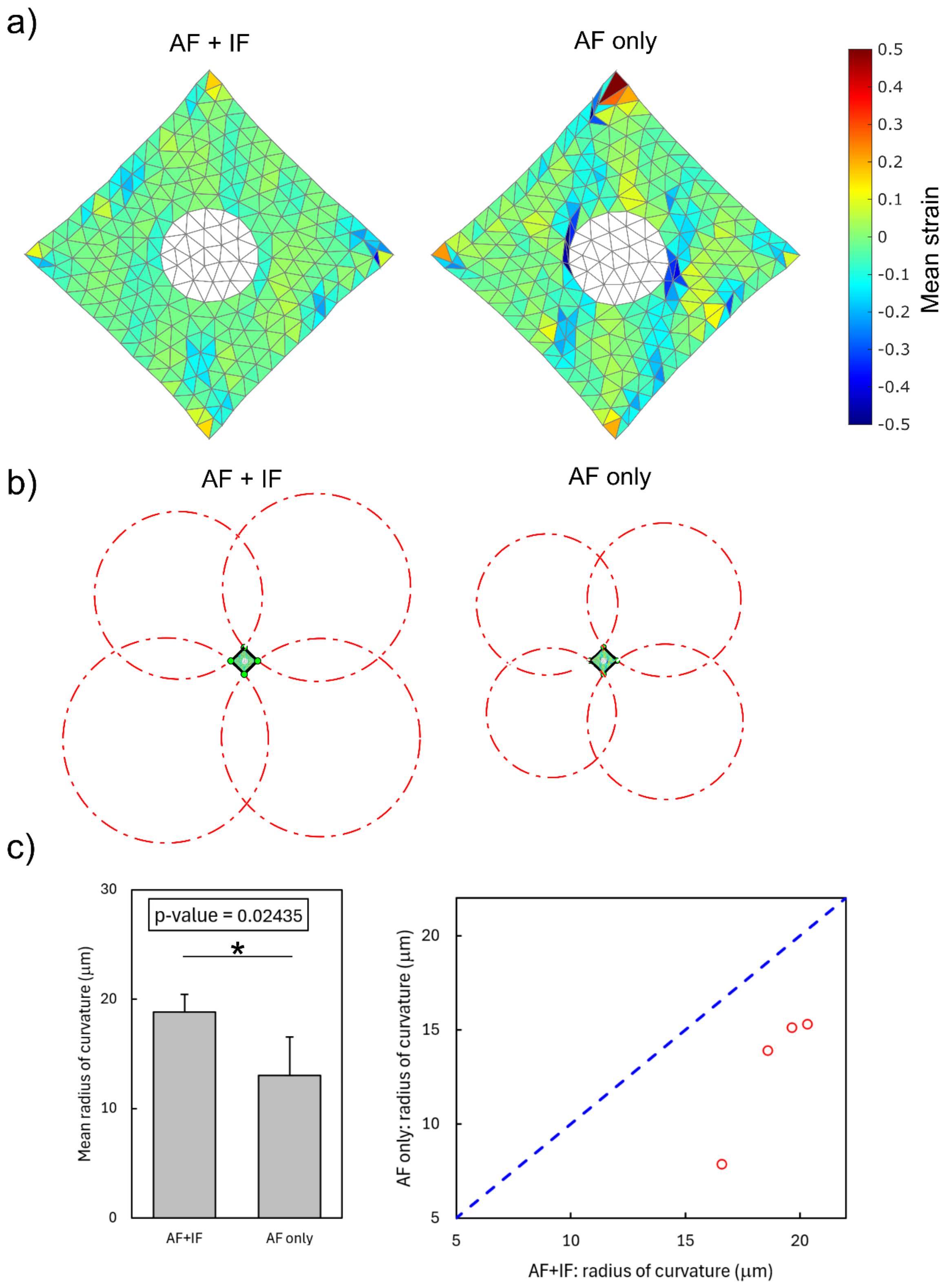

Supplementary Figure 8. Role of intermediate filaments in modulating cellular deformation. a) Mean strain distribution for the case with both AFs and IFs compared to the condition with AFs only. The absence of IFs results in elevated compressive and tensile strains across the domain. b) Fitted circles (red dash-dot lines) through membrane segments for both conditions, used to quantify local curvature. c) Comparison of membrane curvature. Left: Mean radius of curvature across all segments shows a significant reduction when IFs are removed ($p = 0.024$). Right: Scatter plot comparing the radius of curvature for individual membrane segments between the two conditions. The blue dashed line represents the identity line ($y = x$). These results highlight the role of IFs in stabilising cell shape and resisting large deformations. (Error bars represent SD. *$p < 0.05$. *p*-values were calculated using Student's t-test.)

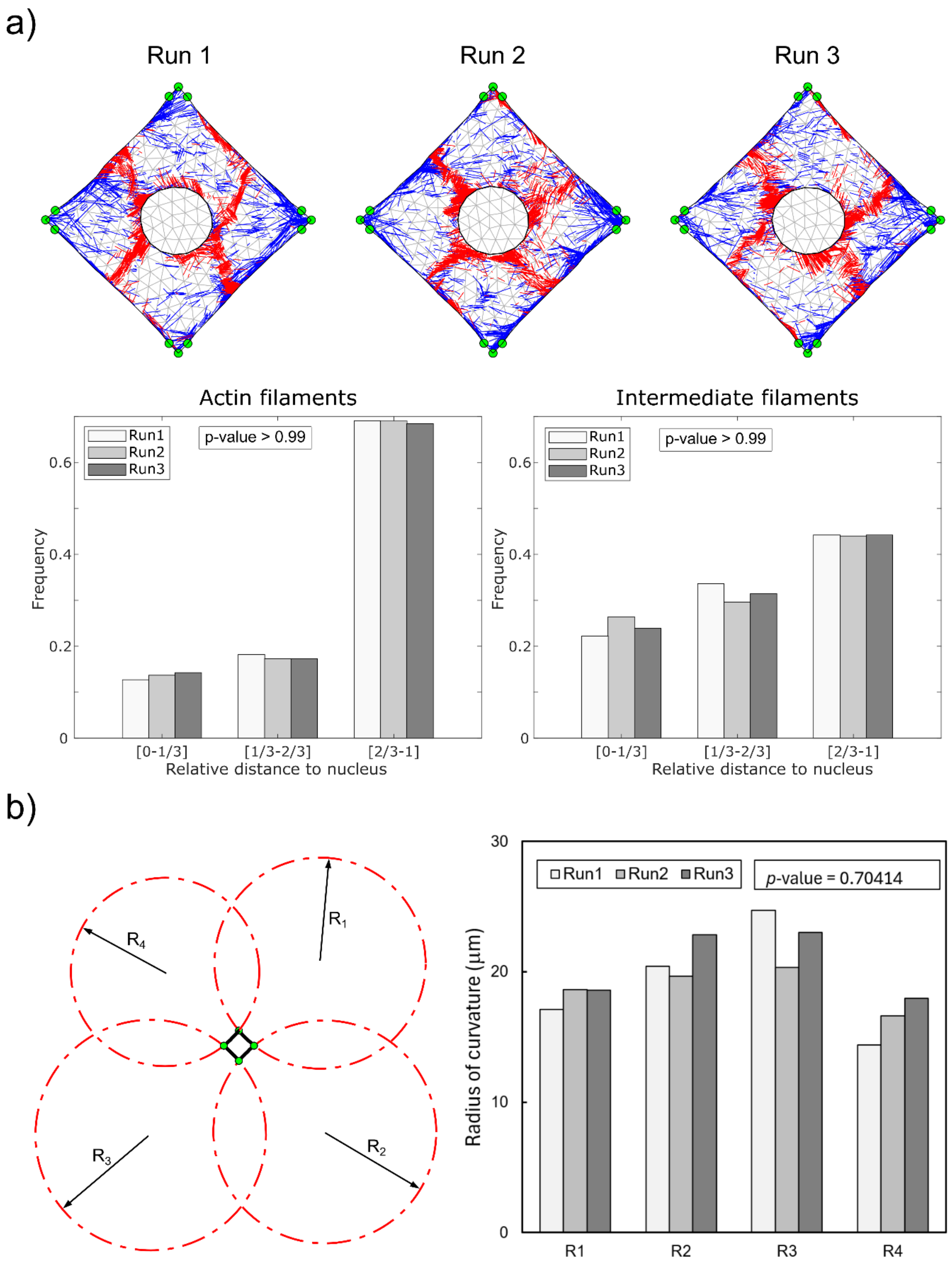

Supplementary Figure 9. Assessment of model reproducibility across independent simulations with different random initial conditions. **a)** Top row: Final distribution of actin filaments (AFs, blue) and intermediate filaments (IFs, red) across three simulation runs. Bottom row: Quantification of filament localisation relative to the nucleus. For both AFs and IFs, high p-values ($p > 0.99$) indicate that the spatial distributions are highly consistent across runs, demonstrating the robustness of the model to stochastic variability. **b)** Left: Representative fitted circles (red dash-dot lines) used to calculate local membrane curvature. Right: Radius of curvature values measured for four membrane segments ($R_1 - R_4$) across the three runs. The high p-value ($p = 0.70$) confirms the model's ability to consistently reproduce global cell shape and membrane curvature. (Statistical analysis was performed using one-way ANOVA.)

References

[S1] Deshpande, V.S., R.M. McMeeking, and A.G. Evans. 2006. A bio-chemo-mechanical model for cell contractility. Proc. Natl. Acad. Sci. 103: 14015–14020.

[S2] Barzegari, A., Y. Omidi, A. Ostadrahimi, V. Gueguen, A. Meddahi-Pellé, M. Nouri, and G. Pavon-Djavid. 2020. The role of Piezo proteins and cellular mechanosensing in tuning the fate of transplanted stem cells. Cell Tissue Res. 381: 1–12.
